# Barriers and facilitators to smartwatch-based prehabilitation participation among frail surgery patients: a qualitative study

**DOI:** 10.1186/s12877-024-04743-6

**Published:** 2024-02-02

**Authors:** Savanna Kerstiens, Lauren J. Gleason, Megan Huisingh-Scheetz, A. Justine Landi, Daniel Rubin, Mark K. Ferguson, Michael T. Quinn, Jane L. Holl, Maria Lucia L. Madariaga

**Affiliations:** 1 Department of Surgery, Biological Sciences Division, University of Chicago, Chicago, IL USA; 2 Department of Medicine, Section of Geriatrics & Palliative Medicine, Biological Sciences Division, University of Chicago Medicine, Chicago, IL USA; 3 Department of Anesthesia and Critical Care, Biological Sciences Division, University of Chicago Medicine, Chicago, IL USA; 4 Department of Medicine, Section of General Internal Medicine, University of Chicago, Chicago, IL USA; 5 Department of Neurology, Biological Sciences Division, University of Chicago, Chicago, IL USA

**Keywords:** Frailty, Prehabilitation, Smartwatch, Surgery, Exercise, Older adult

## Abstract

**Background:**

For older, frail adults, exercise before surgery through prehabilitation (prehab) may hasten return recovery and reduce postoperative complications. We developed a smartwatch-based prehab program (BeFitMe) for older adults that encourages and tracks at-home exercise. The objective of this study was to assess patient perceptions about facilitators and barriers to prehab generally and to using a smartwatch prehab program among older adult thoracic surgery patients to optimize future program implementation.

**Methods:**

We recruited patients, aged ≥50 years who had or were having surgery and were screened for frailty (Fried’s Frailty Phenotype) at a thoracic surgery clinic at a single academic institution. Semi-structured interviews were conducted by telephone after obtaining informed consent. Participants were given a description of the BeFitMe program. The interview questions were informed by The Five “Rights” of Clinical Decision-Making framework (Information, Person, Time, Channel, and Format) and sought to identify the factors perceived to influence smartwatch prehab program participation. Interview transcripts were transcribed and independently coded to identify themes in for each of the Five “Rights” domains.

**Results:**

A total of 29 interviews were conducted. Participants were 52% men (*n* = 15), 48% Black (*n* = 14), and 59% pre-frail (*n* = 11) or frail (*n* = 6) with a mean age of 68 ± 9 years. Eleven total themes emerged. Facilitator themes included the importance of providers (right person) clearly explaining the significance of prehab (right information) during the preoperative visit (right time); providing written instructions and exercise prescriptions; and providing a preprogrammed and set-up (right format) Apple Watch (right channel). Barrier themes included pre-existing conditions and disinterest in exercise and/or technology. Participants provided suggestions to overcome the technology barrier, which included individualized training and support on usage and responsibilities.

**Conclusions:**

This study reports the perceived facilitators and barriers to a smartwatch-based prehab program for pre-frail and frail thoracic surgery patients. The future BeFitMe implementation protocol must ensure surgical providers emphasize the beneficial impact of participating in prehab before surgery and provide a written prehab prescription; must include a thorough guide on smartwatch use along with the preprogrammed device to be successful. The findings are relevant to other smartwatch-based interventions for older adults.

**Supplementary Information:**

The online version contains supplementary material available at 10.1186/s12877-024-04743-6.

## Background

Frailty is a risk factor for older patients undergoing thoracic surgery and is associated with increased length of hospital stay, hospital discharge to post-acute and long-term care facilities, and loss of independence [1–3]. Importantly, frailty is a dynamic phenomenon that can be altered by physical activity, nutritional, and psychological support [4, 5]. Exercise pre-habilitation (prehab) before lung surgery has been shown to improve recovery [6], return to autonomy, and functional capacity [4] while reducing pulmonary complications [7]. Guidelines from the American College of Surgeons “Strong for Surgery” and the National Institute of Aging promote walking or engaging in aerobic and resistance exercises for 30 minutes a day for at least 2 weeks [8, 9]. Both patients and clinicians agree that prehab is beneficial [10]. Despite this, challenges remain in the clinical implementation of prehab [5, 7] and patient participation can be as low as 5 to 12% in routine clinical practice [6, 10, 11].

Health technology, such as mhealth apps and wearable devices, can provide clear instructions and prompts for physical activity [12–14], and can be leveraged to overcome barriers to participation. Indeed, smartwatches have been shown to help improve patient pre-conditioning before and after lung surgery [15]. We developed a smartwatch-based prehab program that uses a Wi-Fi-independent smartwatch (Apple Watch) to deliver a customized physical activity application (app) (BeFitMe) for older, frail patients. The BeFitMe™ smartwatch was developed using the behavior change wheel [16] and provides, encourages, and tracks self-guided at-home exercise for frail older adult patients. The app includes behavior-change notifications and exercise recommendations to increase patient engagement, and records step count and self-reported exercise time (minutes) to support clinicians providing exercise management. We conducted a pilot study to assess uptake of physical activity and the app in thoracic surgery patients [17]. Despite high acceptability of the Apple Watch by patients, actual completion of the recommended physical activity was limited.

Evidence suggests that older adults face significant and unique barriers to technology-assisted prehab programs, such as confusion when using a mobile device, disinterest, lack of confidence, lack of experience in using technology, and lack of technology design that addresses older adult user needs [18]. Better understanding of patient-related needs when using a smartwatch for prehab is needed [12, 14]. Furthermore, there is limited information about the barriers and facilitators to adoption and sustainability of smartwatch-based prehab programs [3, 5, 10, 19]. This study sought to gather older adult patient perspectives about the barriers and facilitators of engaging in physical activity and exercise before thoracic surgery and to identify the factors that would facilitate patient uptake of a smartwatch-based prehab program (BeFitMe).

## Methods

### Study design, setting, and participants

We conducted a descriptive, qualitative study [20] to assess patient perceived factors influencing prehab participation among older adults. The study was conducted at a thoracic surgery clinic at a single, urban, academic medical center and was approved by the University of Chicago Institutional Review Board (IRB22–1679). Eligibility criteria included patients aged ≥50 who were either scheduled to have thoracic surgery or already had thoracic surgery within 2 months. Patients were identified from the electronic medical record. Verbal consent was obtained. Demographic and frailty assessment (Fried’s Frailty Phenotype [21]) were collected as baseline. Participants then underwent a semi-structured interview (< 30 minutes) by telephone by a single researcher (SK). Interviews were recorded and transcribed.

Patients were enrolled and data were gathered until saturation was achieved, whereby no new themes were generated from the data [22]. An initial round of interviews was conducted and served to refine the interview guide, which was used to conduct a second round of interviews. The initial and refined interview guides are in Supplemental File A. Participants in both the initial and second round of interviews are included in the analysis. No participants took part in both rounds of interviews.

### Theoretical framework

The study was situated with the constructivist paradigm, which acknowledges that there is not one objective truth; rather, multiple realities exist based on everyday social interactions and experiences [23]. We employed this framework to help researchers recognize that past experiences and perspectives play a role in interpretation of new information and how pre-existing knowledge can influence the analysis of generating new ideas (themes). Multiple conversations between the coders (SK and MLM) took place to ensure researcher reflexivity.

Interviews were guided by The Five “Rights” of Clinical Decision Support (1) right information, (2) right person, (3) right time, (4) right channel, and (5) right format] from the Healthcare Information and Management Systems Society “Improving Outcomes with Clinical Decision Support: An Implementer’s Guide” [24, 25]. The Five “Rights” were applied to develop interview questions about prehab implementation (Table 1).

**Table 1:** Framework guiding interview questions

**Right Information**	What do patients need to know about the impact of frailty on surgery outcomes and value of prehab? What “messages” would engage and motivate patients to participate in prehab?
**Right Person**	Who do patients need support from to engage in prehab? Who do patients expect/want to engage/motivate them about prehab?
**Right Time**	When should a provider “bring up” information on preparing for surgery and participating in prehab?
**Right Channel**	What “channels” or tools (e.g., paper hand-out, device, in-person with clinician) do patients want/need to be informed of prehab benefits and tasks? What channels do patients want/need to help stay engaged in prehab and the BeFitMe prehab program.
**Right Format**	What formats (e.g. layout, font, colors, set up) are preferred/needed by patients to facilitate engagement in the BeFitMe prehab program?

To understand patient barriers and facilitators to engaging in exercise before thoracic surgery, we asked questions about what matters to patients before surgery and what information they would like to receive before surgery during a preoperative visit, for example “What specific things would you hope to learn about as far as how to prepare for surgery?”. To identify necessary features to promote and increase patient uptake in the BeFitMe prehab program, we asked specific questions about using an Apple Watch for prehab and what would facilitate adherence to the BeFitMe prehab program (Supplemental File A).

### Researcher reflexivity

While gathering the patient perspectives, SK and MLM engaged in rigorous reflexivity processes during several meetings to discuss and assess the impact of their individual experiences, contexts, and knowledge on data analysis and interpretation [26]. The two team members reviewed transcripts separately, and created memos on noteworthy themes, which were then discussed in depth and further refined during an iterative process. Decisions about specific coding and thematic analysis were addressed during the reflexive processes by selecting relevant codes and keywords.

### Data analysis

Recorded interviews were transcribed verbatim by uploading the audio file to Microsoft Word’s transcribe function and then verified by SK prior to analysis, including removal of all identifying information. Data were coded and analyzed using MAXQDA (VERBI software, 2022, Berlin, Germany), following three steps:

#### Step 1: coding

Transcripts were read, independently, by two researchers (SK, MLM) using open coding, whereby codes emerged from the data. Coder reliability between the two coders was assessed using an open source reliability calculation tool [27, 28], with 82% reliability achieved during the first round of coding and 92% with the second round of coding. The research team engaged in reflexivity processes and had several team meetings to review coding and reach consensus.

#### Step 2: generation of themes

Two researchers (SK, MLM) examined participants’ responses that were coded to uncover common perceptions. Deductive coding was performed initially to sort participants responses according to the three major domains based on the interview questions [(1) information before surgery, (2) barriers to prehab, (3) facilitators to prehab] and then inductive coding was performed to develop the final themes within each domain and iterate on existing codes [29]. Concepts generated from the initial round of interviews enabled the researchers to refine the interview guide and add important questions based on the initial specific perspectives. The generation of common perceptions from the second round of interviews followed the same process, where two researchers (SK, MLM) engaged in dialogue and an open coding process to categorize participant perceptions.

#### Step 3: identifying the five “rights” among the themes

A frequency count of common perceptions and statements was derived across each round of interviews. Each common perception was sorted into a theme and then discussed until consensus was reached on themes. Axial coding was then conducted where the researchers identified each theme related to a specific aspect of The Five “Rights” of the Clinical Decision Support Framework.

## Results

### Participants

We interviewed 29 thoracic surgery patients of whom 52% were men (*n* = 15), 48% Black (*n* = 14), and 59% pre-frail (*n* = 11) and frail (*n* = 6), with a mean age of 68 ± 9 years (Table 2). Eight participants completed the interview preoperatively and 21 participants completed the interview postoperatively. Every participant completed the full interview, and the average length of the interviews was 16 ± 5 minutes.

**Table 2:** Patient demographics (*N* = 29)

Characteristics	No. of Patients (%)
Gender:	
Male	15 (52%)
Female	14 (48%)
Age: Mean ± SD, y	68 ± 9 (range 53–85)
Race:	
White	13 (45%)
Black	14 (48%)
Asian/Pacific Islander	2 (7%)
Frailty Status:	
Frail	6 (21%)
Pre-Frail	11 (38%)
Not Frail	9 (31%)
Not screened	3 (10%)

### Themes

Eleven themes and 11 sub-themes were identified. Themes were grouped into the three domains [(1) Information before surgery, (2) barriers to prehab, (3) facilitators to prehab]. A summary of themes and important considerations that the BeFitMe protocol should include to maximize patient adoption are presented in Table 3. Tables showing all themes and common perceptions regarding the information important to older patients in a preoperative assessment, barriers to exercise and the BeFitMe prehab program, and facilitators to exercise and the BeFitMe prehab program can be found in Supplemental File B. The number and percentage of respondents whose responses mapped to each theme are also reported (Supplemental File B).

**Table 3:** Summary of main themes

**Right Information**	Participants identified the right information as the beneficial impact prehab can have on their recovery, quality of life, and overall health.
**Right Person**	Participants identified the clinician as the right person to discuss and prescribe prehab.
**Right Time**	Participants identified early visits (initial visit or subsequent visits to discuss treatment options) as the best time to learn about how to prepare for surgery and the effects of prehab.
**Right Channel**	Participants identified several channels to promote prehab engagement; 1.) prehab prescriptions, 2.) handouts with person-specific recommendations, and 3.) the Apple Watch as a motivational tool for exercise.
**Right Format**	Participants identified a preprogrammed and set-up smartwatch as the proper format for smartwatches to help mitigate the technology barrier for less technologically savvy adults.

### DOMAIN: information before surgery

#### Theme 1: life after surgery

When asked what information is important to learn before surgery, every participant discussed the importance of learning how the surgery will affect their life postoperatively, and most participants also identified learning about the recovery period as important (e.g., “I wanted to know from the doctor what my recovery time would be like. And I just wanted to know more than anything the recovery and you know what kind of activity I could do” (Table 3). Furthermore, participants articulated that knowing if the surgery will improve their quality of life was important to learn about during a preoperative visit (e.g., “I’m hoping that whatever they’re taking care of is gonna change my life for the better”). This theme was well aligned with the “right information” with regard to implementation of the BeFitMe program.

#### Theme 2: ways to communicate

Participants commented that clear and comprehensive clinician communication during the preoperative visit impacted their ability to remember important information about preparing for surgery (e.g., “Give me the key points and why these key points are important, for me to reference in my mind. Because the only thing I got at the age of 70 is words. If you understand what I’m saying”). For some participants, personal investment of the clinician in the care plan was crucial for their own preparation for surgery (e.g., “The only thing that I, how do I say it, that I would like for the doctor is to be more interested”). Clinician investment is well aligned with the “right person”. Additional clinician ways of communication, such as providing patients with physical handouts or written notes or instructions, were often identified as beneficial ways to help patients prepare for surgery and are aligned with the “right channel” with regard to implementation of the BeFitMe Program.

#### Theme 3: preparing for surgery

Participants stated the importance of learning how to prepare for surgery (e.g., “It’s always good to get information about what to what you need to do to prepare for surgery like this”), and in some instances, participants also stated that gaining access to this information early on, such as during the initial visit or upon scheduling the surgery, could help alleviate fear and anxiety (e.g., “I suspect that that would be part of the conversation that you would be having when you find out you’ll need surgery”) (Table 3). Nutrition and exercise were often mentioned as important topics that participants wanted to learn about in order to prepare for surgery (e.g., “Actually having some guidance on eating and drinking and the things that you should be doing ahead of time. What do they want you to do, more exercise or you know, start exercising? You know that would be a good idea but that guidance would be very helpful”). This theme is well aligned with the “right time and right information” with regard to implementation of the BeFitMe program.

### DOMAIN: barriers to prehabilitation

#### Theme 4: pre-existing conditions and fatigue

Eleven participants identified pre-existing conditions, such as symptoms and pain from current illnesses or fatigue, as barriers to doing daily exercise before surgery. Additionally, a few participants mentioned their disinterest in exercise (e.g., “No, I’m just lazy. I don’t do, well exercising at home”), which we identified as a psychological barrier. This theme is aligned with the need to provide the “right information” with regard to implementation of the BeFitMe program.

#### Theme 5: need for additional support

Need for additional support to engage in prehab, such as family support or a gym membership was identified (e.g., “If whoever is helping me to exercise is not serious about me doing it, why should I exercise with them”). This barrier is aligned with “right person” with regard to implementation of the BeFitMe program. Not all patients will have additional support from family, friends, or other care partners.

#### Theme 6: patient choice

Seven participants stated that engaging in exercise is the patient’s choice. These participants expressed skepticism toward other patients following through with the program (e.g., “not sure it [the prehab program] would really be effective because people who don’t want to exercise don’t exercise”). Furthermore, some participants indicated that being told to exercise is a standard recommendation from doctors, and that it takes motivation to follow through and increase activity (e.g., “It has to be self-motivated, within yourself you have to decide I need to get up and get moving so that I can have more energy and it’s something you have to decide within yourself that you are going to do”). This barrier is aligned with the “right person and right information” with regard to implementation of the BeFitMe program.

#### Theme 7: technology barriers

Five participants identified specific technology barriers to the BeFitMe program. However, a few participants reported that the Apple Watch might be a barrier for older patients who are not technologically savvy (e.g., “You know somebody that’s not as techie you know might be overwhelmed by it”). This barrier is aligned with the “right channel” with regard to implementation of the BeFitMe program. Additionally, some participants stated a fully set-up smartwatch that required little use would help uptake and overcome some of the technology barriers (e.g., “I’m not familiar for how easy they are to use but if you have something that’s preloaded and it’s like ‘OK this one is for you’, when you present it and it’s just something they wear and it you know it’s like well it knows time of day and that’s going to tell you your progress throughout the day you know. Do they need to know the technology? No, not really”). This was aligned with the “right format” for BeFitMe implementation.

### DOMAIN: facilitators to prehabilitation

#### Theme 8: BeFitMe program as enjoyable and motivation for exercise

Participants believed the BeFitMe program would be enjoyable and a good motivator for exercise, with several mentioning that the ability to track and monitor physical activity through the smartwatch would be a facilitator for participation. Further, the Apple Watch, itself, was identified as a point of interest (e.g., “I think that people would be interested in them, in that idea I think you know, I mean, there’s a lot of interest in Apple watches to begin with”). This theme is aligned with the “right channel” for the BeFitMe program. Many participants also identified that the “standalone” aspect of the watch was a facilitator for participation (e.g., “Yeah, the standalone watch would definitely, I think it’s huge because I almost feel like it defeats the purpose if you gotta have a phone with you … and then I mean the convenience is huge, you know slapping on your wrist”). This theme is aligned with the “right format” with regard to implementation of the BeFitMe program. Notifications from the BeFitMe Apple Watch app were also viewed positively by many participants who believed that the notification would be a motivational reminder to engage in exercise.

#### Theme 9: extra motivation for engaging in physical activity

Participants expressed the need for extra motivation for exercise before surgery from their clinician, (e.g., “Without the proper motivation, you know, people can tell but what they can tell you a lot of things and that’s just not going to happen”). Some participants commented on the importance of guidance about how to exercise (e.g., “Maybe be more specific on the things that you [the patient] needs to do, other than ‘just keep active.”). Participants also identified some tools, such as a device, a prescription, a checklist, or equipment, as supportive facilitators for exercise (Table 3)(e.g., “I like the idea of a prescription of actually, you know, versus a recommendation where I could take it or leave it”), which is aligned with the “right information and right format” with regard to implementation of the BeFitMe program.

#### Theme 10: relating physical activity to health

A clinician was often identified by participants as the right person to encourage prehab participation (Table 3), with a key facilitator being the clinician relating exercise to positive health outcomes (e.g., “So I would want to say that hey, by from what you did in this moment or weeks leading up to surgery, this is what we expect you to see at the end of all, you know, we expect, you know, this is probably improved it. We expect you’re out of the hospital half a day earlier or than you would have been.”). A few participants identified using fear as a way to encourage participation (e.g., “I’ve got to do this or I’m not going to be able to have surgery. It was fear, or no choice.”). This theme is aligned with the “right information” with regard to implementation of the BeFitMe program.

#### Theme 11: need for individualized support and variety of activities

Participants suggested that individualized support for older patients would help overcome the technology barrier of the smartwatch, such as providing smartwatch training and different types of use (e.g., “What about giving them like a piece of paper? Because you know, you need to give instruction to this elderly person on how to plug in to recharge and you win.”). Participants also stated that engaging in shared-decision making about types of exercise that they could perform and how the watch could be used may facilitate participation (e.g., “know how a person feels about themselves and what they’re willing to do, and then you can begin on small tasks maybe bowling, maybe it might be walking around the neighborhood, you know it could be ping pong, it could be anything but let them have something to say in terms of what they may want to do”). This theme is aligned with the “right channels, formats, and information” to consider when implementing the BeFitMe program.

## Discussion

This study provides some important insights into factors that may influence older patient engagement in a prehab program of physical activity or exercise before surgery, as well as barriers and facilitators to participation in a prehab program that uses a smartwatch. Participants mainly perceived the BeFitMe prehab program as positive and identified the Apple Watch, clinician communication, and guidance on exercise as key facilitators. Our findings also identified some aspects of exercise before surgery and the BeFitMe prehab program that were not suitable for all patients, such as those who are disinterested in exercise or are not technologically savvy enough to use an Apple Watch. These findings align with the Five “Rights” of Clinical Decision-Making and identify key factors that need to be addressed during implementation of the BeFitMe prehab program.

Participants clearly articulated a desire for information about prehab well before surgery. Key facilitators to introducing a prehab program include the clinician being invested in the patients’ care plan and providing clear messaging about how exercise before surgery can positively impact their recovery and post-surgical quality of life. These comments are similar to prior work which shows that providing information early about the immediate benefits of exercise can increase the acceptability of engaging in exercise before surgery by older adults [30]. This study also identifies the importance of motivators, such as individualized support, written instructions, exercise prescriptions, and the convenience of and public interest in Apple Watches, to increase uptake in the BeFitMe prehab program. Providing patients with activity suggestions and a wearable tracker can have a substantial impact on exercise engagement, as older adults have reported these additional features can encourage intrinsic motivation for exercise [30, 31].

While the Apple Watch as a tool for prehab engagement was mostly perceived as a facilitating tool, it was also perceived as barrier for non-technologically savvy patients. For example, the participants that expressed negative attitudes toward the watch (5/29) were 70 or older, while every participant aged younger than 70 years old expressed positive attitudes toward the Apple Watch as a facilitator for exercise. Yet, participants also provided suggestions to overcome this barrier. Having prior experience with smartwatches was not a requirement for this study, and some participants expressed that prior experience with a smartwatch is not required for the BeFitMe program if it is set up specifically for older patients as a standalone, Wi-Fi independent smartwatch. Participants also suggested providing thorough instructions and training on smartwatch use, BeFitMe notifications on the smartwatch, and follow up to further overcome the technology barrier. In fact, prior work has shown that follow-up phone counseling can improve older adults’ adoption of prehab with a wearable device [32]. By providing the patient with the smartwatch and clear instructions on use, and by having the smartwatch completely set-up and formatted for easy use, the technology barrier for older adults could potentially be overcome.

A frail older patient’s baseline health and wellness can influence their perceptions of and adherence to a prehab program (3). While responses by participant frailty status did not differ for most themes, pre-frail and frail participants identified the need for more support to engage in exercise before surgery. A majority of frail participants (5/6) expressed the need for extra motivation to engage in exercise, which suggests a potential benefit of the BeFitMe program, as it can provide frail patients with extra motivation. Additionally, 9 out of the 14 participants who expressed the value of individualized support were pre-frail and frail. This is consistent with other studies showing that clinicians need to provide personalized information and tailor the physical activity recommendations and goals for each patient [33]. Indeed, even small increases in physical activity among more sedentary older adults can improve health outcomes [32, 34]. Optimizing person-centered goal setting (e.g., setting stainable goals based on frailty status) and engaging in shared decision making about what constitutes physical activity may improve older adult participation. Most frail participants (4/6) said that the most important information to learn before surgery is how it will impact their recovery and life after surgery. This sheds light on the importance of clinicians highlighting the potential benefits of participating in the BeFitMe program on a patient’s recovery.

Overall, participants expressed the importance of clinicians (right person) clearly explaining the importance of prehab (right information) early before surgery (e.g., preoperative visit) (right time), which are key factors to participation in the BeFitMe program. Offering a preloaded and set up (right format) Apple Watch (right channel) were also identified as facilitators to participation in a prehab program for older patients (Table 3). From a wider perspective, participants’ positive views suggest that the overall approach to BeFitMe using an Apple Watch has the potential for achieving greater uptake and use than other prehab programs that require patients to have experience with wearable devices, have a compatible smartphone, or have internet connectivity. For BeFitMe to be successful, the many identified facilitators need to be robustly implemented to overcome barriers and increase patient uptake.

### Limitations

This study has several limitations. While we engaged in iterative reflexivity during data collection, bias may have been imposed during analysis since only two researchers coded and analyzed the data. Additionally, the study was not designed to show whether participants’ responses differed depending on their age, clinical condition, the type of surgery received, or pre- versus post-operative status. While the Five Rights of Clinician Decision Making is an established framework, alternative approaches to quality improvement and qualitative evaluation may reveal different findings. Lastly, our findings may not be generalizable to other surgical settings with different patient populations.

## Conclusion

The results of this study identify a wide range of factors that facilitate older, frail patient participation in an exercise prehab program using a smartwatch. Participants reported the importance of knowing the impact and benefit of a prehab program on their health and recovery and endorsed the motivational value of a smartwatch to encourage actual participation in prehab before surgery. The future BeFitMe implementation protocol must ensure surgical providers emphasize the beneficial impact of participating in prehab before surgery and provide a written prehab prescription. Implementation must also ensure patients are given a thorough guide on smartwatch use along with the preprogrammed smartwatch to be successful. The findings are relevant to other smartwatch-based interventions for older adults. Future work is needed to address barriers, such as lack of interest in technology, lack of interest in exercise, and to explore a tailored approach to prehab targeting individual patient needs.

### Supplementary Information

**Additional file 1.** File contains initial and refined interview guides.

**Additional file 2.** File contains tables showing all themes and common perceptions regarding the information important to older patients in a preoperative assessment, barriers to exercise and the BeFitMe prehab program, and facilitators to exercise and the BeFitMe prehab program.

## Data Availability

All data generated or analyzed during this study are included in this published article [and its supplemental information files]. Any additional data are available from the corresponding author on reasonable request.

## Abbreviations

Prehab: Prehabilitation
UC: University of Chicago
