# Supplementary material for: Barriers and facilitators to smartwatch-based prehabilitation participation among frail surgery patients: a qualitative study
Source: BMC Geriatr. 2024 Feb 2;24:129. doi: 10.1186/s12877-024-04743-6 (PMC10835899; doi:10.1186/s12877-024-04743-6)
Supplement: Supplementary file 1 — Additional file 1. File contains initial and refined interview guides. [file 12877_2024_4743_MOESM1_ESM.pdf]

Barriers and facilitators to smartwatch-based prehabilitation participation among frail surgery patients

Semi-structured Interview Guide

Initial Interview guide: (01/30/2023-2/22/2023)

Introduction and consent:

[Consent template]

Questions:

To learn how patients understand prehab.

1. After you learn you will be having surgery, what kind of topics would you hope to learn about prior to surgery?
2. How would you prefer to learn new information to prepare you for your surgery, such as on prehabilitation?
   - In or during your office visit?
   - A prehab prescription?
   - A helpful paper handout?
   - A phone call after the visit?
   - Watching a video?
     - Would you want to watch the video in clinic, or would you prefer a link to the video so you can access it at home when need be?
   - What do you think of an Apple Watch app as a tracker?

3. Are there any strategies you currently use to make sure you are getting enough sleep, eating well, or getting enough exercise throughout the day?

*"**The next few questions have to do with exercise-based prehabilitation, which is like rehab but it happens before surgery."**

4. How would prehabilitation, or routine physical activity before surgery, impact a patient? What are the obstacles to prehabilitation?

5. How could your doctor help to make it clear that increasing activity is a necessary step before surgery?
   - Recommendations of exercises?
   - Written prescription of prehabilitation regimen?

6. What are the barriers, if any, that you have to doing prehabilitation?

To understand what will encourage patients sign up and fully participate in prehab.

7. In an ideal world what would type of prehabilitation would you like to do before surgery?

- When and where would you like to perform prehabilitation?

8. What type of support would you need to stay on track with prehabilitation?
   - Prehab prescription / paper handout?
   - 1:1 In-person guide?
   - 1:1 guide over zoom?
   - Equipment?
   - Transportation?
   - Beftime?
   - Apple watch?
   - Exercise log?
     - o Does the weekly checklist seem like a good motivator?

9. How do you think you would implement these instructions into your daily life in the weeks leading up to your surgery?

Describe BeFitMe prehab program

10. Have you ever used a smart watch before?
    - Would you want to?

11. How would you feel about responding to notifications on this watch every day?

12. Would you be comfortable with someone from the thoracic department reaching out to you weekly or biweekly to see how your progress is going?

13. If you did prehabilitation, what kind of progress would you like to feel or see?
    - How would you like to learn of your progress?
    - What kind of goals would you set for yourself.

Second Interview guide: (04/20/2023-06/15/2023)

Introduction and consent:

[Consent template]

Questions:

To learn what matters most to patients when learning they will need surgery and how the clinical team can help them remember the important information.

1. When you are told you'll need surgery, a lot of information is covered, how would/do you go about remembering the information your doctor tells you?
   a. What would help you remember all the information?
   b. How would you use the information to prepare for surgery?

2. What specific things would you hope to learn as far as how the surgery will impact your life?

3. What specific things would you hope to learn about as far as how to prepare for surgery?

To learn patient perspectives of performing daily exercise and prehab

4. What does exercise mean to you or how would you define exercise?

5. In learning what you can or can't do before surgery, what would you think if your doctor told you that you should perform 20 minutes of physical activity a day leading up to your surgery?
    a. Would knowing that it will benefit you during and after the surgery be enough to motivate you, or are there specific impacts you want to hear?

    b. Would knowing exercise before surgery can help you bounce back to your baseline after surgery encourage you / change your mindset?

6. Why do / or do you not exercise?

7. What are your main barriers to doing physical activity before surgery?

8. How do you develop a mindset to do daily exercise, especially if you feel sick?
    a. What are ways to change a routine?

9. What type of support would be most beneficial coming from your surgeon?

To learn patient perceptions of Apple Watch/BeFitMe prehab program

***Describe BeFitMe prehab program**

10. What are your initial thoughts on this exercise program?
    a. What are the negatives to using Apple Watch?
    b. Why wouldn't you use it?
    c. In what ways is the Apple Watch better than a smartphone alone, if at all?

11. In what ways can I assist you with the Apple Watch that would make participating in the program easy/appealing?
    a. How do you think I can help the older generation, who may not be as tech savvy, with the Apple Watch prehab program?

12. What kinds of benefits do you want to know about?
    a. Do you want to know numbers, percentages, statistics of your progress in the program? Or the benefits/better outcomes that the program may have?

13. If you participated, what kind of follow-up would you like from us?

14. What kind of notification is helpful and how often?
    a. If you don't like notification, what would you like instead?
