# Supplementary material for: Barriers and facilitators to smartwatch-based prehabilitation participation among frail surgery patients: a qualitative study
Source: BMC Geriatr. 2024 Feb 2;24:129. doi: 10.1186/s12877-024-04743-6 (PMC10835899; doi:10.1186/s12877-024-04743-6)
Supplement: Supplementary file 2 — Additional file 2. File contains tables showing all themes and common perceptions regarding the information important to older patients in a preoperative assessment, barriers to exercise and the BeFitMe prehab program, and facilitators to exercise and the BeFitMe prehab program. [file 12877_2024_4743_MOESM2_ESM.docx]

| **Supplemental Table 1. Information Before Surgery** | | | |
| --- | --- | --- | --- |
| **Theme** (Sub-themes) | *“*Specific Beliefs*”* | Five “Rights” Domains | N=29 (%) that  contained theme |
| **Life after surgery** | “Is it going to make my life better what, whatever remaining years I got, or is it going to be more difficult because I'm doing these surgeries.” | Right Information | 29 (100%) |
| Recovery Timeline | “I was very interested how the post recovery is going to look like.” | Right Information | 13 (45%) |
| **Ways to communicate** | “Handouts would be perfect. That would be the easiest way, just give me what they said and I'll just go.” | Right Channel | 19 (66%) |
|  | “When the doctor first explained some things to me, he actually wrote it and drew a picture and I made a little chart of things that were that he thought was important for me to know and that was very helpful because I'm kind of a visual learner.” | Right Person | 12 (41%) |
| Comprehensive Messaging | “If my questions are answered by the doctors that I asked without, you know being basic, and coming straightforward to me with the answers for the questions that I asked, with good answers that I can rely on.” | Right Person | 14 (48%) |
|  | “Putting something in writing is always the best for me because then I have a document that I can refer to about specific the specifics of the surgery.” | Right Channel | 14 (48%) |
| Providers Being Personally Invested | “I think that they need somebody to be paying attention to them you know, really paying attention to whatever their anxieties are, and I think that once they would feel that somebody was really interested in what's happening to them, that which I think would come again through conversation, I think that they would be more inclined to do what's required of them whatever physical activity is suggested or whatever, because by then there would be some kind of trust set up and they would they would know that that's what they needed to do.” | Right Person | 13 (45%) |
| **Preparing for Surgery** | “The day before surgery where they told me what to expect and all that it was very helpful, but again, I would have loved to have gotten that information to help alleviate all anxieties and the fears Ohh, maybe a week or so before, you know, to to know what to expect.” | Right Time | 13 (45%) |
| Nutrition Before Surgery | *“*If there are any dietary restrictions prior to surgery that I should be adhering to.” | Right Information | 13 (45%) |
| Exercise Before Surgery | *“*What do they want you to do, more exercise or you know, start exercising? You know that would be a good idea but that guidance would be very helpful.” | Right Information | 13 (45%) |

| **Supplemental Table 2. Barriers to Exercise and to Participating the BeFitMe Prehab Program Before Surgery** | | | |
| --- | --- | --- | --- |
| **Theme** (Sub-themes) | *“*Specific Beliefs*”* | Five “Rights” Domains | N=29 (%) that  contained theme |
| **Pre-existing Conditions and Fatigue** | “Well the simple answer is when I walk much I'm face to face with the emphysema and my breathing problems. So, I don't take that walk every day because it's very unpleasant and depressing when all the sudden you realize your emphysema is really affecting your movement.” | Right Information | 11 (38%) |
| Psychological Barriers | “Just don't just not doing it, there's no, no particular reason. Just not doing it like I should.” | Right Information | 7 (24%) |
| **Need for Additional Outside Support** | “It’s difficult for a person to change some of their habits you know, and you need a support system, and you need people to encourage you.” | Right Person | 9 (31%) |
| **Patient choice** | “I look at it from standpoint of me being 81 years old and knowing my body, I basically all these years so I basically know what I need to do with my body and dictating to me and telling me I got to do 20 minutes you know it's my it's my decision if I want to or not.” | Right Information | 7 (24%) |
|  | “Anytime I go to any doctors they say do you exercise do you exercise and you say Oh my need to get busy, but you know people don't do that.” | Right Person | 7 (24%) |
| **Technology Barriers** | “What I don't like about the Apple Watch thing is that you're talking about a group of people that are not attached to those things like young people.” | Right Channel | 5 (17%) |

| **Supplemental Table 3. Facilitators to Exercise and to Participating the BeFitMe Prehab Program Before Surgery** | | | |
| --- | --- | --- | --- |
| **Theme** (Sub-themes) | *“*Specific Beliefs*”* | Five “Rights” Domains | N=29 (%) that  contained theme |
| **BeFitMe Prehab Program as enjoyable and Motivation for exercise** | “That's a good program. That's a that's a good reminder to have, like you say, Apple watches give you reminders or give you advice, right so that definitely that's very important.” | Right Channel | 24 (83%) |
|  | “I can totally see the benefit and it would give you some nice biofeedback right away too.” | Right Information | 13 (45%) |
|  | “Everything is right there and they make the numbers on it and everything big enough so you know that should be an easy, I guess that could be a good sale for the older generation.” | Right Format | 8 (28%) |
| Notifications from BeFitMe Would be a Good Reminder | “That's actually, I think that's great cause I mean we love, the older I think the old generation love reminders and no I think that's fine.” | Right Format | 16 (55%) |
|  | *“*It's good to have the notification to say you need to do something more than this. That’s a big help of course.” | Right Channel | 16 (55%) |
| Provide a Sense of Achievement | *“*When you're walking and you're tracking how much you walk, it definitely helps you potentially walk a little more than you might otherwise.” | Right Format | 13 (45%) |
|  | “I think we all like that instant gratification so we can see those numbers go up as far as steps or mileage or whatever, that's motivation, that Apple Watch is definitely motivation, yeah.” | Right Information | 9 (31%) |
| **Extra Motivation for Engaging in Physical Activity** | “I just say present some data that shows that this you know this this person I feel was more successful with surgery because of this amount of walking or this amount of biking or this amount of exercise.” | Right Information | 23 (79%) |
|  | “I think you know an encouraging call going ‘Hey, I saw that you're actually doing the exercises and getting what you need’ You know that that would be beneficial. ” | Right Person | 10 (34%) |
| “Prescription” for Exercise Before Surgery | “That would have been good. I mean, like I said, it gets you on the frame of mind that you know, you need to, not even just before surgery, but after surgery, you should you just want to just walk, a mile or two a day.” | Right Time | 14 (48%) |
|  | “I would definitely, if it was suggested by any doctor yes absolutely, anything I'd be told to do, I would absolutely do it.” | Right Person | 12 (41%) |
|  | “A written prescription of exercise would be nice.” | Right Format | 11 (38%) |
| **Relating Physical Activity to Health** | “I think just telling it would probably not be the best way to do it, but to go, hey, all right, if you do this, then it's going to shorten your recovery and really help get you out of the hospital faster, then that would probably be a pretty good motivation.” | Right Information | 19 (66%) |
|  | “I think the mental part of a surgery approaching is a harder task than the physical part of the surgery and so I think maybe for me, it's knowing you've got to do this” | Right Time | 7 (24%) |
| Knowing Benefits of Participating in BeFitMe | “You need to know the benefits of having it and you know what the results would show. I think that that would be a good thing.” | Right Information | 10 (34%) |
|  | “I think it would be fine and if I knew it was related to you know overall healthcare situation that I guess I'd say I would find it probably some degree of comforting to know that even though I know it's just an automated thing that some something is paying attention.” | Right Information | 8 (28%) |
| Fear of Illness | “I think the only thing that would really get some people, and I'm going to use my husband for an example, to change habit into exercising more is fear…. So it was like I've got to do this or I'm not going to be able to [have surgery] it was fear, or no choice.” | Right Information | 7 (24%) |
| **Need for Individualized Support or Variety of Activities** | “I think maybe that program would have to be geared towards the person you know, knowing what the person's limitations are.” | Right Person | 14 (48%) |
|  | “Give people a couple of choices on any given day so that they feel more empowered and have more control.” | Right Channel | 14 (48%) |
|  | “You'd have to explain more gently, probably what it's for and what the responsibility would be in having to use the technology.” | Right Information | 8 (28%) |
|  | “Well, one way will be maybe make the numbers or the letters, the fonts a little bigger because you know we have the vision sometimes is not as good as young people.” | Right Format | 8 (28%) |

| **Supplemental Table 4. Responses according to surgery status - Preoperative and Postoperative** | | |
| --- | --- | --- |
| **Theme and Sub-theme** | **Preoperative**  **Participants (%)**  **(N= 8)** | **Postoperative**  **Participants (%) (N= 21)** |
| Life After Surgery | 7/8 (88%) | 16/21 (76%) |
| Handouts as Preferred Methods of Communication | 5/7 (63%) | 14/21 (67%) |
| Learning about Nutrition to Prepare for Surgery | 6/8 (75%) | 7/21 (33%) |
| Learning about Exercise to Prepare for Surgery | 5/8 (63%) | 8/21 (38%) |
| Pre-existing Conditions or Fatigue | 4/8 (50%) | 7/21 (33%) |
| Need for Additional Outside Support | 4/8 (50%) | 5/21 (24%) |
| Patient Choice | 1/8 (13%) | 6/21 (29%) |
| Technology Barriers | 1/8 (13%) | 4/21 (19%) |
| BeFitMe Prehab Program as enjoyable and Motivation for exercise | 7/8 (88%) | 17/21 (81%) |
| Extra Motivation for Engaging in Physical Activity | 7/8 (88%) | 15 /21 (71%) |
| Relating Physical Activity to Health | 5/8 (63%) | 15/21 (71%) |
| Need for Individualized Support or Variety of Activities | 4/8 (50%) | 5/21 (24%) |
